# Dual and opposing roles of EIN3 reveal a generation conflict during seed growth

**DOI:** 10.1016/j.molp.2021.11.015

**Published:** 2022-02-07

**Authors:** Juliane Heydlauff, Isil Erbasol Serbes, Dieu Vo, Yanbo Mao, Sonja Gieseking, Thomas Nakel, Theresa Harten, Ronny Völz, Anja Hoffmann, Rita Groß-Hardt

**Affiliations:** 1 University of Bremen, Centre for Biomolecular Interactions Bremen (CBIB), Leobenerstrasse 5, 28359 Bremen, Germany; 2 ZMBP, University of Tübingen, Auf der Morgenstelle 32 72076 Tübingen, Germany

**Keywords:** seed size, EIN3, ethylene biosynthesis, fertilization, generation conflict, synergid disintegration

## Abstract

Seed size critically affects grain yield of crops and hence represents a key breeding target. The development of embryo-nourishing endosperm is a key driver of seed expansion. We here report unexpected dual roles of the transcription factor EIN3 in regulating seed size. These EIN3 functions have remained largely undiscovered because they oppose each other. Capitalizing on the analysis of multiple ethylene biosynthesis mutants, we demonstrate that EIN3 represses endosperm and seed development in a pathway regulated by ethylene. We, in addition, provide evidence that EIN3-mediated synergid nucleus disintegration promotes endosperm expansion. Interestingly, synergid nucleus disintegration is not affected in various ethylene biosynthesis mutants, suggesting that this promoting function of EIN3 is independent of ethylene. Whereas the growth-inhibitory ethylene-dependent EIN3 action appears to be encoded by sporophytic tissue, the growth-promoting role of EIN3 is induced by fertilization, revealing a generation conflict that converges toward the key signaling component EIN3.

## Teaser

Seed size can have a critical influence on grain yield. We found that the transcription factor EIN3 has two opposing functions during seed development: a seed growth-promoting role and an ethylene-dependent seed growth-inhibiting role. The two functions are exerted by different tissues, suggesting an EIN3-mediated generation conflict that modulates seed development.

## Introduction

Seeds are a key food source for humans and animals and an important basis for biofuel production, making them an important target of breeding programs. In flowering plants, the main components of the seed, the embryo and the embryo-nourishing endosperm, result from fertilization of an egg and an adjoining central cell. The two sperm necessary for this so-called double fertilization are delivered by a single pollen tube, which finds its way to the female gametes due to a sophisticated guidance system (Johnson et al., 2019; Hater et al., 2020; Hafidh and Honys, 2021). Short-range pollen tube attraction is mediated by two egg–cell-adjoining synergid cells, which secrete cysteine-rich peptides (Higashiyama et al., 2001; Márton et al., 2005; Okuda et al., 2009; Takeuchi and Higashiyama, 2012; Meng et al., 2019; Zhong et al., 2019) and mediate the discharge of two sperm from the pollen tubes arriving in the female gametophyte (Huck et al., 2003; Rotman et al., 2003; Amien et al., 2010). Pollen tube arrival is accompanied by programmed cell death of the first synergid. The disintegration of the second synergid and concomitant termination of pollen tube attraction require gamete fusion. This is evidenced by the work of Beale et al. and Kasahara et al., who have shown that incomplete fertilization or the delivery of gamete-fusion-defective sperm suppresses disintegration of the second synergid, resulting in the attraction of supernumerary pollen tubes (Beale et al., 2012; Kasahara et al., 2012).

Synergid disintegration and the establishment of a pollen tube block is a multiphasic process. It involves (i) fertilization-induced cleavage of LURE1 by the egg-secreted endopeptidases ECS1 and ECS2 (Yu et al., 2021), (ii) dilution of LURE by fusion of the synergid with the central cell in a process that requires central cell fertilization (Maruyama et al., 2015), and (iii) synergid nucleus disintegration (Völz et al., 2013; Maruyama et al., 2015). The last step is regulated by fertilization-independent seed–Polycomb Repressive Complex 2 as well as the transcription factors EIN3 and EIL1, which are components of the ethylene response pathway (Maruyama et al., 2013, 2015; Völz et al., 2013). In plants defective for any of these factors, the nucleus of the second synergid remains intact. We, in addition, have shown that synergid-derived nuclei initiate endosperm marker gene expression after fertilization and take on the cell-cycle regime of the endosperm in plants defective for EIN3 (Völz et al., 2013). Consequently, EIN3 prevents the formation of a maternal, haploid synergid-derived endosperm fraction and internuclear heterogeneity. Interploidy crosses have previously suggested that changes in the paternal-to-maternal genome ratio within endosperm nuclei have important implications for seed development, with a relative increase in paternal gene copies promoting seed development, whereas an increase in maternal copies results in smaller seeds (Scott et al., 1998). When analyzing the developmental implications of the internuclear heterogeneity, we found that EIN3 has dual and opposing roles during seed development: while EIN3 in the sporophytic tissue represses seed development, EIN3 signaling activated by fertilization promotes it through the selective degeneration of a synergid nucleus. Our results thus uncover an EIN3-mediated generation conflict that modulates seed development.

## Results and discussion

### Ploidy differences in the seed can be detected in the zygote stage

**Figure 1:**
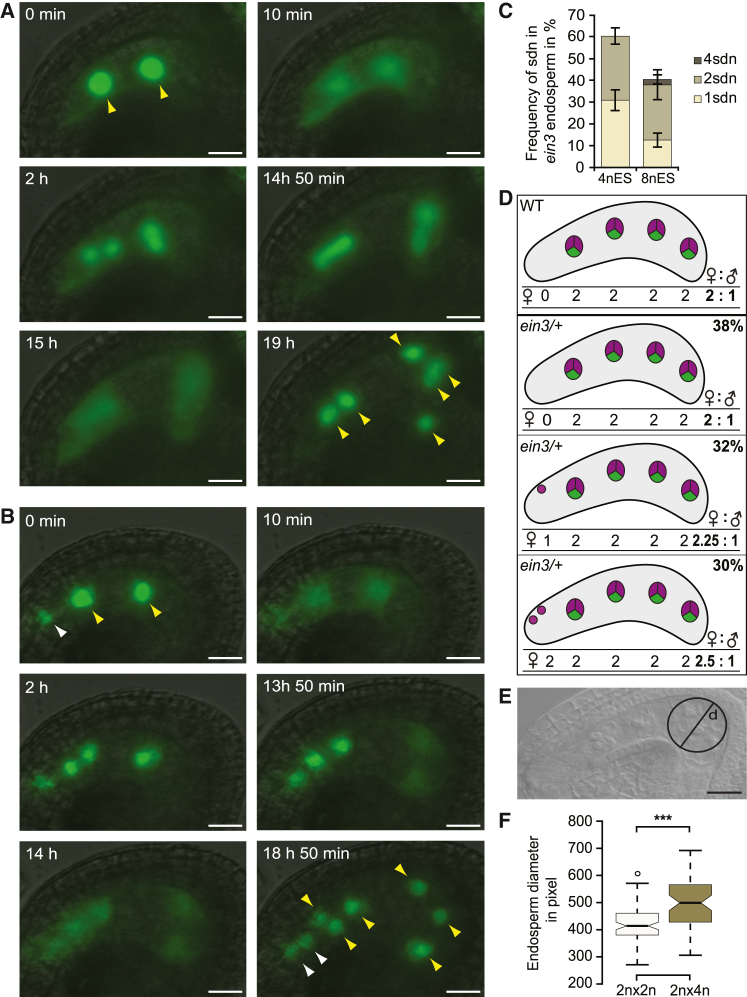
Ploidy differences in the seed can be detected at the zygote stage. **(A and B)** Live-cell imaging of young *ein3*/− × wild-type seeds without **(A)** or with **(B)** sdn. The combinatorial multicolor marker FGR 7.0 confers green fluorescence to sdn and endosperm nuclei. Endosperm nuclei, yellow arrowheads; sdn, white arrowheads. See also Supplemental Videos 1 and 2. **(C)** Frequency of one, two, or four sdn in ein3/− × wild-type seeds at the four- (4nES) and eight-nucleate endosperm stage (8nES) (*n* = 136/166 for 4nES/8nES respectively). **(D)** Schematic representation indicating the changes in maternal- (magenta) to-paternal (green) genome ratio in the four-nucleate endosperm of a plant recovered from an *ein3/-* x wild-type cross containing no sdn, one sdn, or two sdn (*n* = 338). **(E)** Wild-type seed in the four-nucleate endosperm stage. Dorsoventral endosperm diameter was determined by superimposing a circle inside the endosperm at its widest point using ImageJ. **(F)** Dorsoventral endosperm diameter of interploidy crosses in wild-type seeds at the four-nucleate endosperm stage (*n* (2*n* × 2*n*) = 84; *n* (2*n* × 4*n*) = 58). Data are the mean ± SEM. Scale bar, 20 μm. Two-tailed Student’s *t*-test: ∗∗∗*p* < 0.001.

We have previously shown that the synergid-derived nucleus (sdn) in *ein3eil1* mutants adopts the fate and division regime of the endosperm, which we also detected in single *ein3* mutant (Figures 1A, 1B, and Supplemental Figures 1, and 2). In addition, the sdn incorporates a paternally introduced molecular marker, indicating an internuclear transfer of molecules from the biparental endosperm to the asexual, maternal nuclei (Völz et al., 2013) (Supplemental Figure 1A–1C). To investigate the developmental implications of the resulting parental heterogeneity on endosperm development, we aimed to analyze early seed development at the four-nucleate stage, when the difference in the parental architecture and the concomitant formal shift in maternal-to-paternal genome ratio between *ein3* and wild-type endosperm is particularly pronounced (Figure 1C and 1D). In a first step, we asked whether young wild-type seeds at the four-nucleate endosperm stage are already susceptible to changes in maternal-to-paternal ratio. We therefore performed an interploidy cross of diploid wild-type plants with a tetraploid wild-type pollen donor and determined the dorsoventral endosperm diameter in the four-nucleate stage (Figure 1E).

We detected a significantly increased endosperm diameter in seeds having inherited twice as many paternal copies (2*n* × 4*n*) (Figure 1F). While we cannot exclude the possibility that increased pollen tube content of 4*n* plants contributed to this effect (Kasahara et al., 2016; Zhong et al., 2017), these data suggest that even young seeds in the zygotic stage are responsive to ploidy changes.

### Manipulation of EIN3 signaling suggests an inhibitory role of synergid-derived nuclei in endosperm expansion

**Figure 2:**
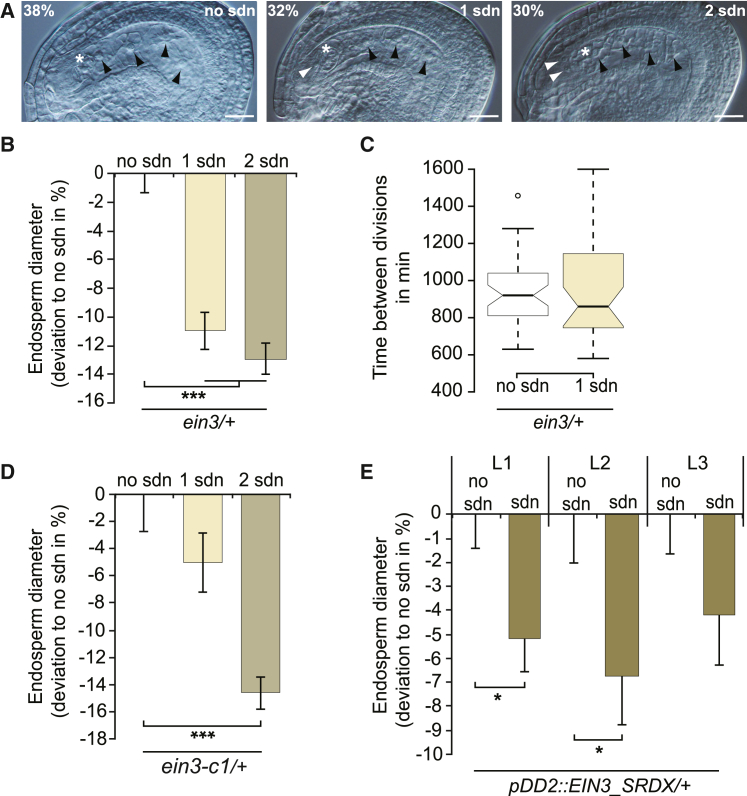
Manipulation of EIN3 signaling suggests an inhibitory role of synergid-derived nuclei in endosperm expansion. **(A)** Representative cleared whole mounts of *ein3*/− × wild-type seeds at the four-nucleate endosperm stage containing no sdn (left), one sdn (middle), or two sdn (right) (*n* = 338). Endosperm nuclei, black arrowheads; sdn, white arrowheads; zygote, asterisk. **(B)** Deviation of dorsoventral endosperm diameter of *ein3*/− × wild-type seeds segregating either one or two sdn at the four-nucleate endosperm stage from the seeds segregating no sdn (*n* = 130/107/101 for no sdn/1sdn/2sdn, respectively). Endosperm diameter of seeds with either one or two sdn is shown relative to endosperm diameter of seeds without sdn. **(C)** Time interval between nuclear disintegration of the two-nucleate and the four-nucleate endosperm stage (*n* = 42/36 for no sdn/sdn). **(D)** Dorsoventral endosperm diameter of *ein3-c1*/− wild-type seeds segregating no, one, or two sdn at the four-nucleate endosperm stage (*n* = 45/70/207 for no sdn/1sdn/2sdn, respectively). Endosperm diameter of seeds with either one or two sdn is shown relative to endosperm diameter of seeds without sdn. See also Supplemental Figure 1D–1F. **(E)** Dorsoventral endosperm diameter of *pDD2::EIN3_SRDX* × wild-type young seeds at the four-nucleate endosperm stage. L1, L2, and L3 indicate independent *pDD2::EIN3_SRDX* lines (*n* (L1) = 81/104; *n* (L2) = 73/16; *n* (L3) = 85/49 for no sdn/sdn, respectively). Data indicate the mean ± SEM. Scale bar, 20 μm. Two-tailed Student’s *t*-test: ∗*p* < 0.05; ∗∗∗*p* < 0.001.

In contrast to interploidy crosses, which shift the ploidy of both endosperm and embryo, the overall maternal-to-paternal ratio in *ein3* mutants is affected only in the endosperm. In addition, *ein3* mutants exhibit a parental internuclear heterogeneity that contrasts with the homogeneous parental ratio characteristic of endosperm nuclei resulting from wild-type or interploidy crosses. To understand whether this idiosyncratic endosperm composition affects endosperm development, we measured dorsoventral endosperm diameter of *ein3* seeds in the four-nucleate stage. We capitalized on our previous finding that the defect of synergid nucleus inheritance is not fully penetrant in *ein3* mutants (Figures 1C and 2A) (Völz et al., 2013), i.e., we were able to compare seeds from the same flowers that either had or had not inherited sdn. We found that endosperm expansion was significantly reduced in *ein3* seeds containing both biparental endosperm and sdn compared with *ein3* seeds with biparental endosperm only (Figure 2B). While this result suggests that sdn negatively affect endosperm expansion, we could not rule out the possibility that development in sdn-segregating ovules was retarded. In fact, interploidy crosses performed by Scott et al. showed that increased maternal copies correlated with delayed mitotic progression and premature cellularization (Scott et al., 1998). To test this hypothesis, we assessed endosperm size dynamics on the basis of live-cell imaging. We introduced a combinatorial multicolor marker, FGR 7.0, into *ein3* plants. FGR 7.0 confers fluorescence to synergids, zygotes, and endosperm (Völz et al., 2013). In addition, we established a protocol for visualization of nuclear dynamics in early endosperm. This allowed us to trace fertilized ovules over a period of 24 h, during which the endosperm underwent up to three mitotic divisions (Figure 1A and 1B; Supplemental Videos 1 and 2). To determine whether early seed development is slowed down in sdn-containing seeds, we used the decondensation of nuclei as a molecular timer. The durations of nuclear disintegration between the two-nucleate endosperm stage and the four-nucleate endosperm stage were comparable, independent of the segregation of sdn (Figure 2C). Together, these results indicate that the size differences are not an artifact introduced by retarded development, but that instead the segregation of sdn correlates with reduced early endosperm expansion.

Supplemental Video 1. Related to Figure 1A: Live-cell imaging of early endosperm nuclei divisions in *ein3*/+ seeds without sdn Pictures were taken every 10 min. The last frame before nuclear decondensation in the two-nucleate endosperm stage was set to 0. Frame numbers are indicated in the video.

Supplemental Video 2. Related to Figure 1B: Live-cell imaging of early endosperm nuclei divisions in *ein3*/+ seeds with sdn Pictures were taken every 10 min. The last frame before nuclear decondensation in the two-nucleate endosperm stage was set to 0. Frame numbers are indicated in the video.

To test whether this effect was indeed causally linked to the *ein3* locus, we generated a CRISPR-induced *EIN3* allele (*ein3-c1*). This allele contains a frameshift insertion at the position of the 495th base, resulting in a premature stop codon after 165 amino acids (Supplemental Figure 1D and 1E). The *ein3-c1* allele exhibits a stronger phenotype than the *ein3* allele with respect to the frequency of sdn-segregating seeds (Supplemental Figure 1F), whereas the overall effect of sdn on endosperm size was comparable (Figure 2D).

The correlation between sdn inheritance and endosperm size is compatible with two conceptually different scenarios: either endosperm expansion and the degeneration of the synergid nucleus are different effects of EIN3-dependent processes operating in the female gametophyte, or the persistence of the maternal haploid synergid nuclei is causally linked with reduced endosperm expansion. To discriminate between the two scenarios, we confined the EIN3-dependent defect to synergids only making use of the SRDX repressor motif (Hiratsu et al., 2003). We have previously shown that expression of the dominant negative *EIN3_SRDX* fusion under the control of the synergid-specific *pDD2* promoter phenocopies the synergid nuclear disintegration defect of *ein3* mutants (Völz et al., 2013). When analyzing the endosperm diameter of different *pDD2::EIN3_SRDX* transgenic lines, we observed substantial phenotypic variations. However, as a common denominator, we found that endosperm size is reduced in the presence of sdn compared with seeds without sdn (Figure 2E). As a control, we expressed the construct after fertilization only in the endosperm using the *AtrBohD* promoter (Völz et al., 2013). This approach did not affect synergid disintegration (*n* = 136/146/203 for wild type/L1/L2), nor did we observe reduced seed size (Supplemental Figure 1G), suggesting that the endosperm does not contribute to the effect in sdn-containing *pDD2::EIN3_SRDX*.

Together, our results indicate that internuclear heterogeneity caused by sdn and reduced endosperm size are causally linked. This also implies that fertilization-dependent synergid nuclei disintegration mediated by EIN3 promotes endosperm expansion. This finding is also in line with and in support of the parental conflict theory, which holds that both parents have different interests in the allocation of resources to a single seed of the same mother plant (Haig and Westoby, 1989).

### Sporophytic EIN3-dependent signaling represses endosperm and seed expansion

**Figure 3:**
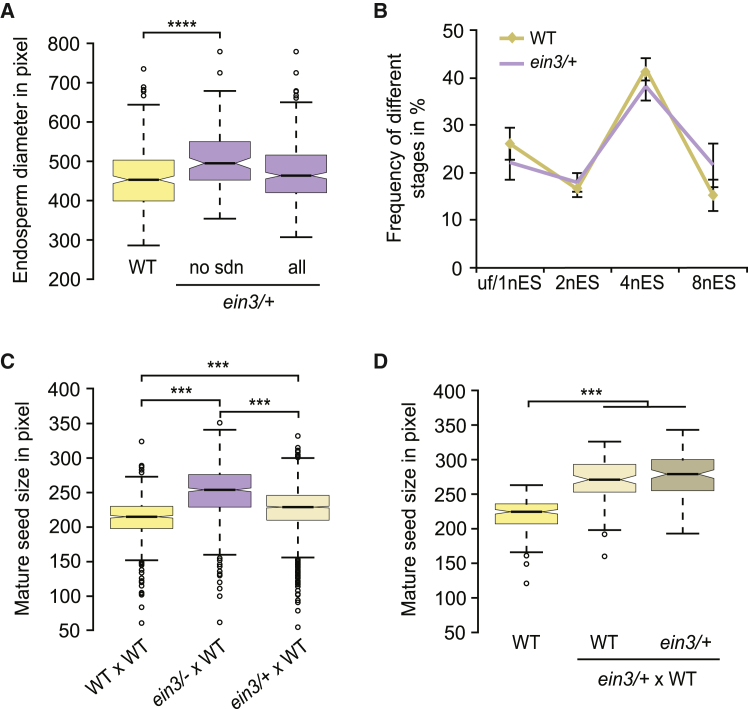
Sporophytic EIN3-dependent signaling represses endosperm and seed expansion. **(A)** Dorsoventral endosperm diameter of wild-type (WT) × WT and *ein3*/− × WT seeds in the four-nucleate endosperm stage. “All” indicates *ein3*/+ seeds of *ein3*/− × WT crosses with and without sdn (*n* = 289/130/338 for WT seeds/ *ein3/+* seeds with no sdn/ all of *ein3/+* seeds). **(B)** Frequency of WT × WT and *ein3*/− × WT seeds in different developmental stages 24 h after pollination: unfertilized and freshly fertilized seeds in the one-nucleate endosperm stage (uf/1nES) and two-, four-, and eight-nucleate endosperm staged seeds (2nES, 4nES, and 8nES, respectively) (*n* = 537/570 for WT/*ein3*/+). **(C)** Mature seed size (*n* = 689/770/2079 for WT × WT seeds/*ein3*/− × WT seeds/*ein3*/+ × WT seeds, respectively). **(D)** Independent experiment showing size of WT × WT and *ein3*/+ × WT mature seeds. Seeds were assigned according to the genotype of their progeny (*n* = 198/109/130 for WT/segregating WT/segregating *ein3*/+, respectively). Two-tailed Student’s *t*-test: ∗∗∗*p* < 0.001 and ∗∗∗∗*p* < 0.0001. See also Supplemental Figure 2.

While young sdn-segregating *ein3* seeds have a reduced endosperm diameter, previous results have reported an increased mature seed size of *ein3* mutants resulting from inhibitory effects of EIN3 on embryo development (Meng et al., 2018). We similarly observed an effect on seed size in both *ein3* and *ein3-c1* alleles (Supplemental Figure 2A–2C). Notably, this effect was evident only when we introduced the mutation through the female (Supplemental Figure 2B). We next asked whether and to what extent a growth-inhibiting effect of EIN3 is observable at an early developmental stage. In fact, we detected an EIN3-dependent growth-inhibiting effect also in early developmental stages, where it is masked by the opposing sdn-dependent effect (Figure 3A). Combining the all, sdn-segregating, and no sdn-segregating *ein3* categories yields an endosperm diameter comparable to that of wild type, while the endosperm diameter in *ein3* seeds without sdn is significantly bigger compared with wild type (Figure 3A). Since there is no significant developmental shift between wild type and *ein3* (Figure 3B), we can exclude that size deviations are caused by different developmental stages, which was further substantiated by live-cell imaging and by analyzing the *ein3-c1* allele (Supplemental Figure 2D–2F).

We next asked whether the repressive effect of EIN3 originated from the gametophyte or the sporophyte. To discriminate between sporophytic and gametophytic function we compared seeds from wild-type, *ein3* homozygous, or *ein3* heterozygous plants pollinated with wild type. While *ein3* heterozygous seeds were smaller than *ein3* homozygous seeds, they were still significantly bigger than wild-type seeds (Figure 3C). This result is compatible with two different scenarios: either EIN3 has a dose-dependent sporophytic effect, which would affect the size of all seeds, or the intermediate seed size results from a mixed filial generation containing smaller wild type segregating and bigger *ein3*/+ segregating seeds. To distinguish between the two scenarios, we followed individual seeds to the seedling stage, where we genotyped them. Our results revealed that all seeds from *ein3* heterozygous plants are significantly bigger than wild type but similar in size independent of the genotype of their embryo (Figure 3D). This result indicates that the EIN3 growth-inhibiting effect is attributable to the sporophytic tissue.

Together, these data indicate that EIN3 has opposing and spatially distinct roles in seed expansion: the EIN3 growth-inhibiting function is regulated by the sporophytic tissue, and this effect is counteracted in early endosperm stages by a growth-promoting EIN3 function, which is mediated by disintegration of the non-receptive synergid nucleus after fertilization. In mature seeds, the effect of sdn appears to become dominated by the growth-inhibiting effect of EIN3, potentially due to the fact that the endosperm is degraded during seed development.

### Ethylene reduction affects the growth-inhibitory but not the growth-promoting function of EIN3

EIN3 is stabilized in the presence of ethylene (Guo and Ecker, 2003; Potuschak et al., 2003). In addition to its fundamental role in many developmental processes and stress responses, the plant hormone has a regulatory role in cell division, cell expansion, and growth (Abeles et al., 1992; Dubois et al., 2018). In light of the opposing effects of EIN3, we next asked whether the growth-promoting and the inhibitory roles of EIN3 equally respond to ethylene.

Ethylene biosynthesis is initiated by the production of S-adenosylmethionine from methionine by S-adenosylmethionine synthase (SAM), which is followed by two additional steps: first, S-adenosyl-L-methionine is converted to 1-aminocyclopropane-1-carboxylic acid (ACC) by aminocyclopropane-1-carboxylic acid synthase (ACS), and second, ACC is converted to ethylene by aminocyclopropane-1-carboxylic acid oxidase (ACO) (Adams and Yang, 1979; Pattyn et al., 2020).

To trace ethylene biosynthesis in time and space, we generated transcriptional and translational reporter lines and analyzed 15 members of the SAM, ACS, and ACO enzyme families involved in ethylene biosynthesis. Twenty-four hours after fertilization, when the endosperm effect of *ein3* was already evident, we detected SAM1 and SAM2 in the sporophyte, endosperm, and zygote; SAM3 in the endosperm and zygote; *pACS2* in the endosperm only; *pACS6* and *pACO2* in the sporophyte only; and *pACO5* in the endosperm of young seeds (Supplemental Figure 3A–3C). This result is also supported by a recent study revealing that ethylene production as well as mRNA abundance of some *ACS* genes gradually increases during seed development (Sun et al., 2020).

**Figure 4:**
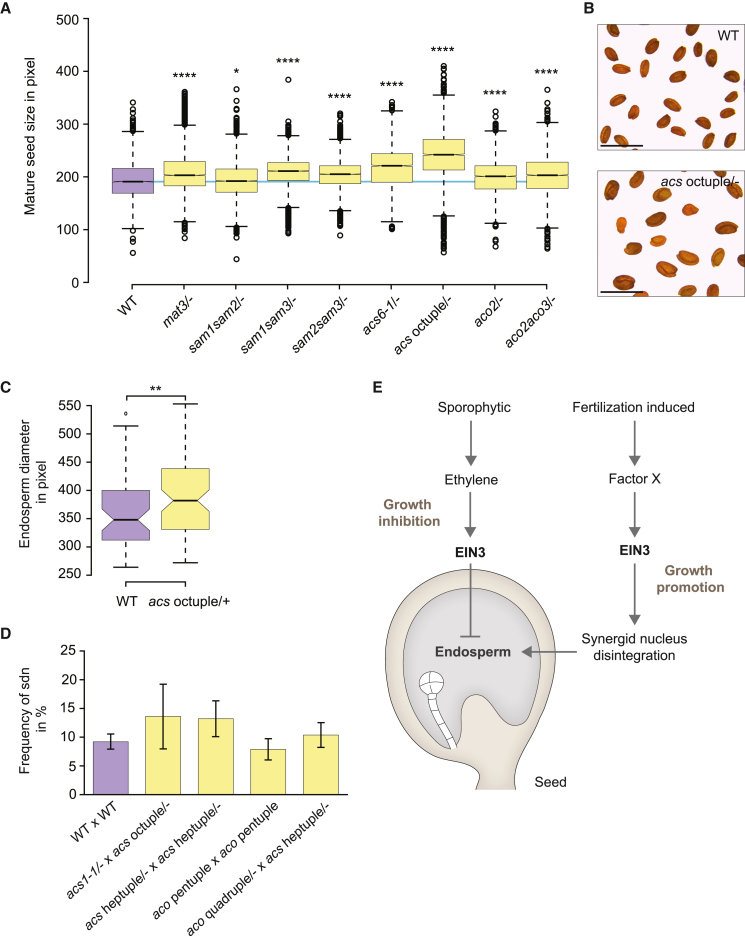
Ethylene reduction affects the growth-inhibitory but not the growth-promoting function of EIN3. **(A)** Dry seed size of different ethylene biosynthetic mutants and wild type (WT) after selfing (*n* = 6508*/*5322*/*4751*/*4736*/*5390*/*2911*/*3453*/*4966*/*5597 for WT*/mat3*/−*/sam1sam2*/−*/sam1sam3*/−*/sam2sam3*/−*/acs6-1*/−*/acs* octuple/−*/aco2*/−*/aco2aco3*/−). The area of each seed collected from 15 individual plants sown at three different times was measured in ImageJ. A boxplot was generated based on individual seed area calculated in pixels. The blue line shows the WT median. **(B)** Dry seeds of WT and *acs* octuple/− plants. Scale bars, 1 mm. **(C)** Dorsoventral endosperm diameter of WT × WT and *acs* octuple/− × WT seeds in the four-nucleate endosperm stage. Seeds showing abnormal development at the micropylar end (see also Supplemental Figure 4B and 4C) are excluded (*n* = 53/79 for WT/*acs* octuple/−). **(D)** Frequency of sdn in the seeds in the four- and eight-nucleate endosperm stages (*n* = 531, 125, 227, 418, 289 for WT × WT, *acs1-1*/− × *acs* octuple/−, *acs* heptuple/− × *acs* heptuple/−, *aco* pentuple × *aco* pentuple, *aco* quadruple/− × *acs* heptuple/−). Two-tailed Student’s *t*-test did not detect significant difference between mutants and WT for sdn frequency. **(E)** Schematic model of dual and opposing roles of EIN3 during seed growth. Two-tailed Student’s *t*-test between WT and mutants: ∗*p* < 0.05; ∗∗*p* < 0.01; ∗∗∗∗*p* < 0.0001. See also Supplemental Figure 4.

We next capitalized on ethylene biosynthesis mutants to address the functional relevance of ethylene production during seed development. It was previously reported that the level of ethylene production in *Arabidopsis* is directly regulated by ACSs (Tsuchisaka et al., 2009) and SAMs (Mao et al., 2015). When analyzing dry seed size of various mutants and mutant combinations targeting *SAM*, *ACS*, and/or *ACO* genes, we detected an increase in seed size compared with wild type (Figure 4A). The effect was particularly pronounced in *acs* octuple seeds in which ethylene production has previously been shown to be strongly reduced (Tsuchisaka et al., 2009) (Figure 4A and 4B). Except for *acs* octuple mutants, which show 49% non-developing/sterile ovules in mature siliques (*n* = 910), all mutants show fertile siliques, indicating that the larger seed size is not due to additional resources freed up by the formation of fewer seeds (Supplemental Figure 4A). On closer inspection of *acs* octuple mutants, we found that a substantial fraction exhibited integument abnormalities and early defects in female gametophyte development (Supplemental Figure 4D), which potentially contribute to a previously described defect of *acs* octuple mutants in pollen tube attraction (Mou et al., 2020). In addition, we observed an increased dorsoventral endosperm diameter in the four-nucleate stage, indicating that seed size deviation initiates early in *acs* seed development (Figure 4C).

We next asked whether synergid degeneration was also susceptible to a reduction of ethylene content. To characterize synergid disintegration in young *acs* octuple seeds, we analyzed cleared whole mounts 1 day after pollination. Interestingly, only 6% (*n* = 105) of the analyzed *acs* octuple seeds showed sdn in the four-nucleate endosperm stage, which is similar to the 4% observed in wild type (*n* = 53). Similar results were obtained when analyzing various combinations of *acs* and *aco* mutant lines, suggesting that synergid disintegration requires no or only a small amount of ethylene (Figure 4D). These results are consistent with recent work by Li et al., which shows that synergid disintegration is not affected in plants fully depleted of ACO function (Li et al., 2021). Our data suggest that synergid degeneration and the concomitant EIN3 growth-promoting function are not or are less responsive to ethylene, indicating that there might be a factor X activating EIN3 signaling after fertilization. Candidates include jasmonic acid (Zhu et al., 2011), salicylic acid (He et al., 2017), or salt (Peng et al., 2014), which have previously been implicated in the regulation of EIN3.

In conclusion, our findings unravel an unexpected dual role of EIN3 during early seed development, a function that is masked in wild-type plants because the effects oppose each other. Intriguingly, the effects are exerted by different tissues representing different generations: while sporophytic tissue represses endosperm and seed expansion in an EIN3-dependent manner, fertilization-triggered synergid disintegration promotes endosperm expansion, thereby ensuring the biparental origin of all endosperm nuclei. Our results, in addition, suggest that the dual and conflicting processes exerted by the two generations differ with respect to ethylene responsiveness: while the depletion of key ethylene biosynthesis genes affects seed expansion, synergid disintegration is not affected, suggesting that the latter process is either fully ethylene independent or sensitive to small traces of ethylene (Figure 4E). Given that ethylene integrates various external and internal stresses, it will be an attractive challenge for the future to determine whether and to what extent endosperm development is amenable to adaptation and how the different functions of EIN3 are regulated.

## Methods

### Plant materials and growth conditions

Seeds of *Arabidopsis thaliana* were sown and stratified at 4°C for 2 days. Stratified seeds were transferred to a Conviron MTPS growth chamber for germination and further growth under long-day conditions (16 h light/8 h dark) at 23°C. Plants were later transferred to 18°C after bolting.

The following plant lines are in the L*er* background: *ein3-1* (referred to as *ein3* in this study), *ein3-1eil1-2* (referred to as *ein3eil1* in this study), *ein3eil1* with *pMEA::NLS_tdTomato* or *pRPS5a::NLS_GFP; pDD2::EIN3_SRDX* (Völz et al., 2013). The *ein3-1eil1-2* double mutant was kindly provided by Richard D. Vierstra. *ein3-1* was crossed out with L*er* wild type. For live-cell imaging, FGR 7.0 lines in the L*er* and *ein3* background were used (Völz et al., 2013). For interploidy assay, 2*n* L*er* and 4*n* L*er* kindly provided by Prof. Dr. Tobias Würschum were used.

Ethylene biosynthetic mutants were obtained from the European *Arabidopsis* Stock Center (NASC) (Nottingham, UK): *sam1* (N573599), *sam2* (N676306), *sam3* (N552289), *mat3* (N519375), *acs6-1* (N16569), *acs* heptuple (N16650), *acs* octuple (N16651), *aco1* (N682904)*, aco2* (N527311), *aco3* (N582132), *aco4* (N514965), and *aco5-2* (N411335). *sam1sam2*, *sam1sam3*, *sam2sam3*, and *aco2aco3*, *aco* quadruple (*aco1*/−*aco2*/−*aco3*/−*aco4*/−), and *aco* pentuple (*aco1*/−*aco2*/−*aco3*/−*aco4*/−*aco5-2*/+) were generated by crossings of the respective single mutants. Col-0 was used as a control.

### Generation of *ein3-CRISPR* line (*ein3-c1*)

All constructs as well as cloning procedures were described previously by Fauser et al. (2014). The protospacer used as a recognition site for the Cas9 nuclease was localized in the exon at position 478–498 of the coding sequence (Supplemental Table 1) and was followed by an AGG protospacer-adjacent motif. The thereby induced mutation has a 1 nt insertion between positions 494 and 495 of the coding sequence and was named *ein3-c1* (Supplemental Figure 1D). This insertion disrupts an NlaIII restriction site important for genotyping and induces a frameshift resulting in a premature stop codon after 165 of 628 amino acids. The *ein3-c1* mutant was outcrossed several times to remove the *CAS9* gene and to reduce off-target mutations. The homozygous mutant was then used for further analyses. L*er* and *ein3* were used as controls.

### Molecular cloning

To generate *pSAMX::gSAMX_tdTomato_tNOS* plasmids, the promoter and genomic loci of *SAM1* (AT1G02500), *SAM2* (AT4G01850), and *SAM3* (AT3G17390) were amplified from the *Arabidopsis* Col-0 DNA library by the respective primers listed in Supplemental Table 1. The promoters digested with AscI and PacI and the genomic fragments digested with PacI and AvrII were subcloned into DR13 plasmid (*pAt5g40260::NLS_tdTomato_tNOS*) (Völz et al., 2013) followed by exchanging *pAt5g40260* with *pSAMX* and *NLS* with *gSAMX*. In addition, to generate *pACSX::NLS_GUS_tNOS* plasmids, the promoters of *ACS2* (AT1G01480), *ACS4* (AT2G22810), *ACS5* (AT5G65800), *ACS6* (AT4G11280), *ACS7* (AT4G26200), *ACS8* (AT4G37770), *ACS9* (AT3G49700), and *ACS11* (AT4G08040) were amplified from the *Arabidopsis* L*er* DNA library by the respective primers listed in Supplemental Table 1. The promoters *pACS2*, *pACS6*, and *pACS11* were digested with AscI and PacI, whereas the promoters *pACS4*, *pACS5*, *pACS7*, and *pACS9* were digested with AscI and PvuI, and the promoter *pACS8* was digested with AscI and XhoI. Afterward, they were subcloned into *pLIS::NLS_GUS_tNOS* (Groß-Hardt et al., 2007), followed by exchanging pLIS with pACSX. Last, to generate *pACOX::NLS_tdTomato_tNOS* plasmids, the promoters of *ACO1* (AT2G19590), *ACO2* (AT1G62380), *ACO3* (AT1G12010), and *ACO5* (AT1G77330) were amplified from the *Arabidopsis* L*er* DNA library by the respective primers listed in Supplemental Table 1. The promoters digested with AscI and PacI were subcloned into DR13 plasmid followed by exchanging *pAt5g40260* with *pACOX*. All plasmids were then transformed into Col-0 plants by floral dip as previously described (Zhang et al., 2006).

### PCR-based genotyping

Genotyping primers are listed in Supplemental Table 2.

### Histology and microscopy

For the analysis of early seed development, the oldest closed flower bud of a given inflorescence was emasculated. One day after emasculation, the flowers were pollinated with wild-type pollen and harvested 24 h later.

For whole-mount clearings flowers were vacuum infiltrated in an ethanol:acetic acid solution (9:1) for 30 min, kept at 4°C overnight, washed for 1 h each with 80% and 70% ethanol, and mounted in chloral hydrate:glycerol:water solution (8:2:1; w:v:v). Cytochemical staining of GUS activity was performed on samples as described previously (Vielle-Calzada et al., 2000). GUS-stained samples as well as cleared whole mounts were then visualized under a Zeiss Axioscope (Zeiss, Oberkochen, Germany) and images were captured by a Canon PowerShot G10 camera. Fluorescence signals were detected by a Leica DMI6000B microscope (Leica Microsystems, Wetzlar, Germany).

### Live-cell imaging

Flowers were used 20 h after pollination to perform live-cell imaging. Pistils were harvested and the two septa were separated by an apical–basal incision alongside the transmitting tract by using an insulin syringe (BD MicroFine). The septum halves with the attached ovules were transferred to an ovule medium modified after Palanivelu et al. (2003): 1 mM MgSO_4_, 4 mM CaCl_2_, 0.01% H_2_BO_3_, 3% PEG 4000, 14.5% sucrose (pH 5.9 adjusted with KOH), and 1.5% NuSieve GTG agarose (Lonza Bioscience). Subsequently the ovules were covered with 200 μl halocarbon oil 700 (Sigma-Aldrich)*.* Live-cell imaging was performed using a Leica DMI6000B microscope (Leica Microsystems, Wetzlar, Germany) equipped with LAS AF version 2.2.1. Ovules in the two-endosperm stage were selected by using the mark and find function. The images were taken every 10 min over a period of 24 h. Four-nucleate endosperm duration was determined by using the time points when the fluorescence signal was still nuclear localized, shortly before nuclear division at the two- and four-nucleate endosperm stages.

### Seed size measurement

Mature seeds were harvested and dried. Dry seeds were scanned by a CanoScan 9000F Mark II in black/white mode with transmitting light and 1200 dpi resolution. Seed area measurement was performed as described previously by using ImageJ (Herridge et al., 2011).

### Data analysis

Datasets were analyzed using Microsoft Excel 2007. Bar charts were created in Microsoft Excel and modified in Adobe Illustrator. Boxplot graphs were generated with BoxPlotR (Spitzer et al., 2014) and modified in Adobe Illustrator. Center lines show the medians; box limits indicate the 25th and 75th percentiles as determined by R software; whiskers extend 1.5 times the interquartile range (IQR) from the 25th and 75th percentiles, and outliers are represented by dots. Crosses indicate the mean. The notches are defined as ±1.58 ∗ IQR/sqrt(*n*) and represent the 95% confidence interval for each median. Statistical analyses were performed in the Analysis ToolPak of Microsoft Excel 2007.

## Funding

We gratefully acknowledge financial support from the 10.13039/100010663European Research Council to R.G. (ERC Consolidator Grant "bi-BLOCK" ID 646644, ERC Proof of Concept Grant "TriVolve" ID 957547).

## Author contributions

Conceptualization, J.H., I.E.S., R.V., and R.G.; methodology, J.H., I.E.S., and R.G.; investigation, J.H., I.E.S., T.H., D.V., Y.M., R.V., S.G., and T.N; visualization, J.H., I.E.S., Y.M., and R.G.; writing – original draft, J.H., I.E.S., and R.G.; writing – review & editing, J.H., I.E.S., and R.G.; resources and funding acquisition, R.G.

## Supplemental information

Document S1. Supplemental Figures 1–4 and Supplemental Tables 1 and 2

Document S2. Article plus supplemental information
