# Supplementary material for: Dual and opposing roles of EIN3 reveal a generation conflict during seed growth
Source: Mol Plant. 2022 Feb 7;15(2):363–71. doi: 10.1016/j.molp.2021.11.015 (PMC8837274; doi:10.1016/j.molp.2021.11.015)
Supplement: Document S1. Supplemental Figures 1–4 and Supplemental Tables 1 and 2 [file mmc1.pdf]

Molecular Plant, Volume *15*

Supplemental information

Dual and opposing roles of EIN3 reveal a generation conflict during seed growth

Juliane Heydlauff, Isil Erbasol Serbes, Dieu Vo, Yanbo Mao, Sonja Gieseking, Thomas Nakel, Theresa Harten, Ronny Völz, Anja Hoffmann, and Rita Groß-Hardt

SUPPLEMENTAL INFORMATION

Dual and opposing roles of EIN3 reveal a generation conflict during seed growth

Juliane Heydlauff[1,3], Isil Erbasol Serbes[1,3], Dieu Vo[1], Yanbo Mao[1], Sonja Gieseking[2], Thomas Nakel[1], Theresa Harten[1], Ronny Völz[2], Anja Hoffmann[1] and Rita Groß-Hardt[1,*]

[1]University of Bremen, Centre for Biomolecular Interactions Bremen (CBIB), Leobenerstr. 5, 28359, Bremen, Germany.
[2]ZMBP, University of Tübingen, Auf der Morgenstelle 32, 72076, Germany.

[3]These authors contributed equally to this work.

*Correspondence: gross-hardt@uni-bremen.de

List of Supplemental Information:

Supplemental Figure 1-4

Supplemental Table 1 and 2

Supplemental movie 1 and 2 legends

SUPPLEMENTAL FIGURES

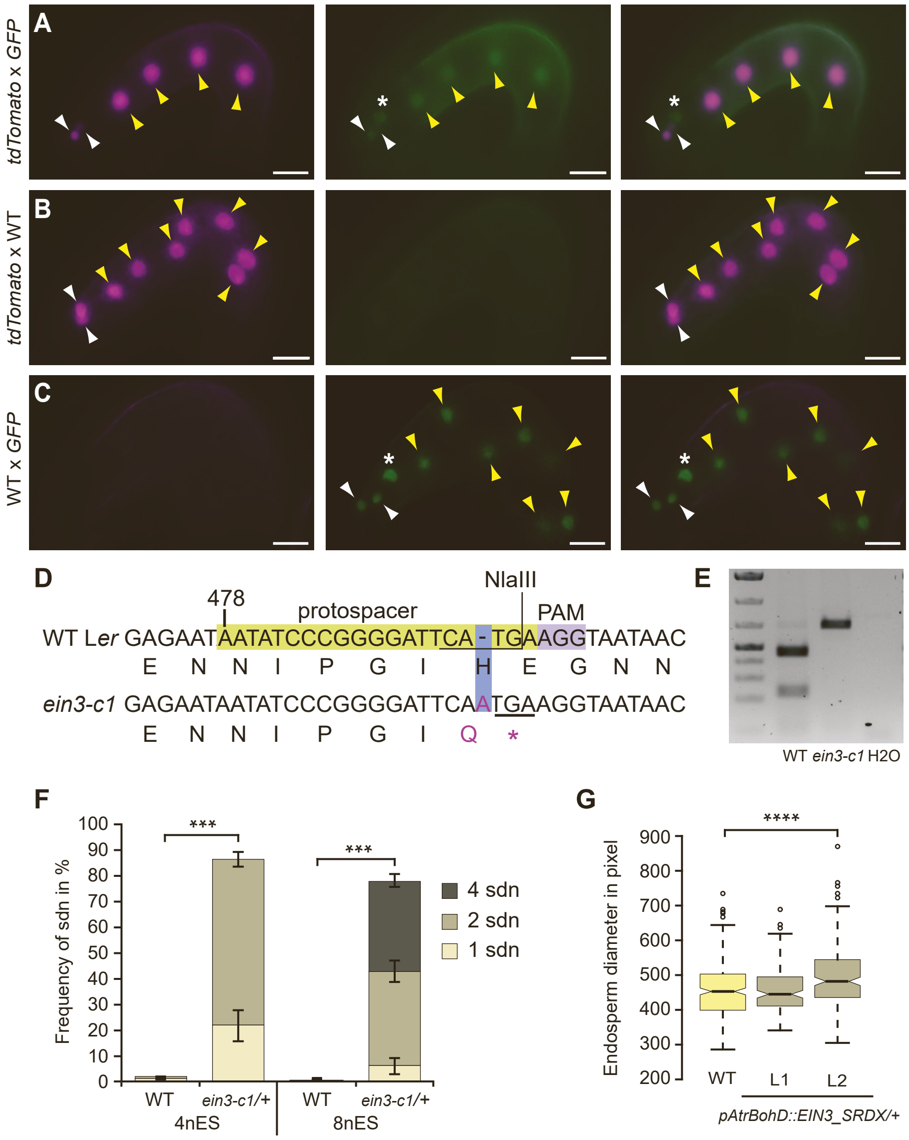

Figure S1: Manipulation of EIN3 signaling suggests an inhibitory role of synergid-derived nuclei in endosperm expansion

(A-C) Incorporation of paternally introduced GFP into sdn. *ein3eil1* seeds carrying both a maternally introduced endosperm marker *pMEA::NLS_tdTomato* expressed in endosperm and sdn, and a paternally introduced ubiquitous marker *pRPS5A::NLS_GFP* expressed in zygote, endosperm and sdn (A), *ein3eil1* seeds carrying only the maternally introduced *pMEA::NLS_tdTomato* marker (B) and wild type (WT) seeds carrying only the paternally introduced *pRPS5A::NLS_GFP* (C). Endosperm nuclei, yellow arrowhead; sdn, white arrowhead; zygote, asterisk. Paternal GFP signal was observed in 90% (n=48) of all tdTomato positive sdn (negative control (B), 6% (n=51)). (D) Frameshift CRISPR/Cas9 generated *EIN3* allele *ein3-c1*. * indicates premature stop codon. (E) PCR and NlaII based detection of the *ein3-c1* allele. WT indicates wild type. (F) Frequency of one, two or four sdn in wild type and *ein3-c1* x WT seeds in the four- (4nES) and eight-nucleate endosperm stage (8nES) (n for 4nES: 261/335 and n for 8nES=292/397 for WT and *ein3-c1*/+ respectively). (G) Dorso-ventral endosperm diameter of wild type and of *pAtRbohD::EIN3_SRDX* x wild type young seeds at the four-nucleate endosperm stage. L1 and L2 indicate independent *pAtRbohD::EIN3_SRDX* lines (n=289/146/203 for WT/L1/L2 respectively). Two-tailed Student's t-test did not detect significant difference between WT and L1 for endosperm diameter. Scale bar, 20 µm. Data indicate mean ± SEM. Two-tailed student's t-test: *** $p < 0.001$ and **** $p < 0.0001$.

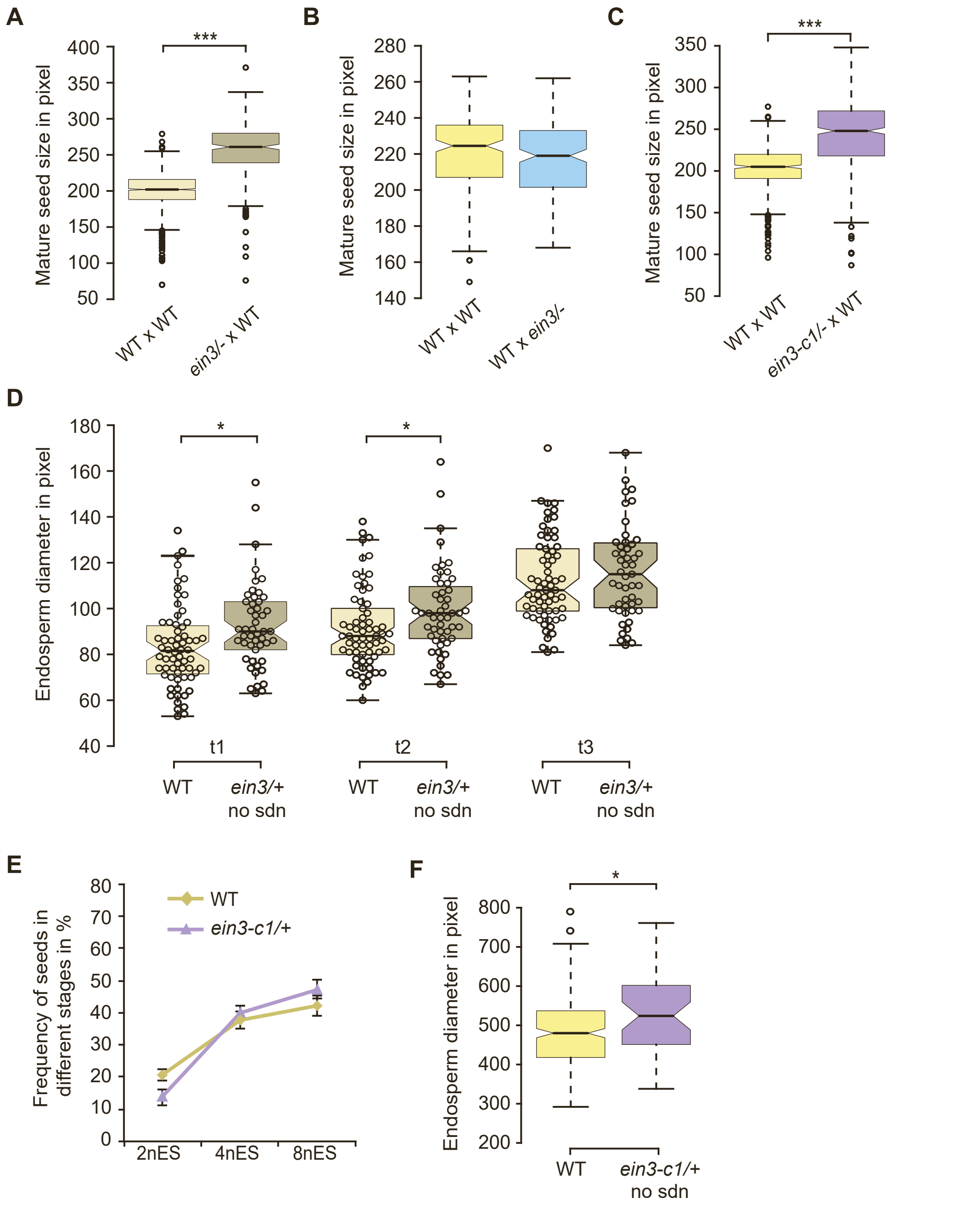

Figure S2: Sporophytic EIN3-dependent signaling represses endosperm and seed expansion.

(A) Size of wild type (WT) x WT and *ein3/-* x WT mature seeds (n=1392/297 for WT/*ein3/+*). (B) Size of WT x WT and WT x *ein3/-* mature seeds (n=198/192 for WT/*ein3/+*). (C) Size of WT x WT and *ein3-c1/-* x WT mature seeds (n=454/481 for WT/ *ein3-c1/+*). (D) Endosperm diameter of WT x WT and *ein3/-* x WT seeds with no sdn extracted from live-cell imaging videos. We used nuclear disorganization as initiation points for mitotic divisions and defined the last picture frame, in which the GFP signal in the two- and four-nucleate endosperm stage was still nuclear localized as t1 and t3, respectively, while the first picture frame in the four-nucleated endosperm stage with condensed GFP signal is defined as t2. (t1; WT, n=64; *ein3/+* n=49, t2; WT, n=66; *ein3/+* n=51 and t3, WT, n=65; *ein3/+* n=51). (E) Frequency of WT x WT and *ein3-c1/-* x WT seeds in different developmental stages 25 hours after pollination: two-, four- and eight-nucleate endosperm stage (2nES, 4nES and 8nES, respectively). (n=691/838 for WT/ *ein3-c1/+*). (F) Dorso-ventral endosperm diameter of WT x WT and *ein3-c1/-* x WT seeds in the four-nucleate endosperm stage. (n=253/45 for WT/ *ein3-c1/+* with no sdn). Two-tailed student's t-test: * $p < 0.05$; *** $p < 0.001$.

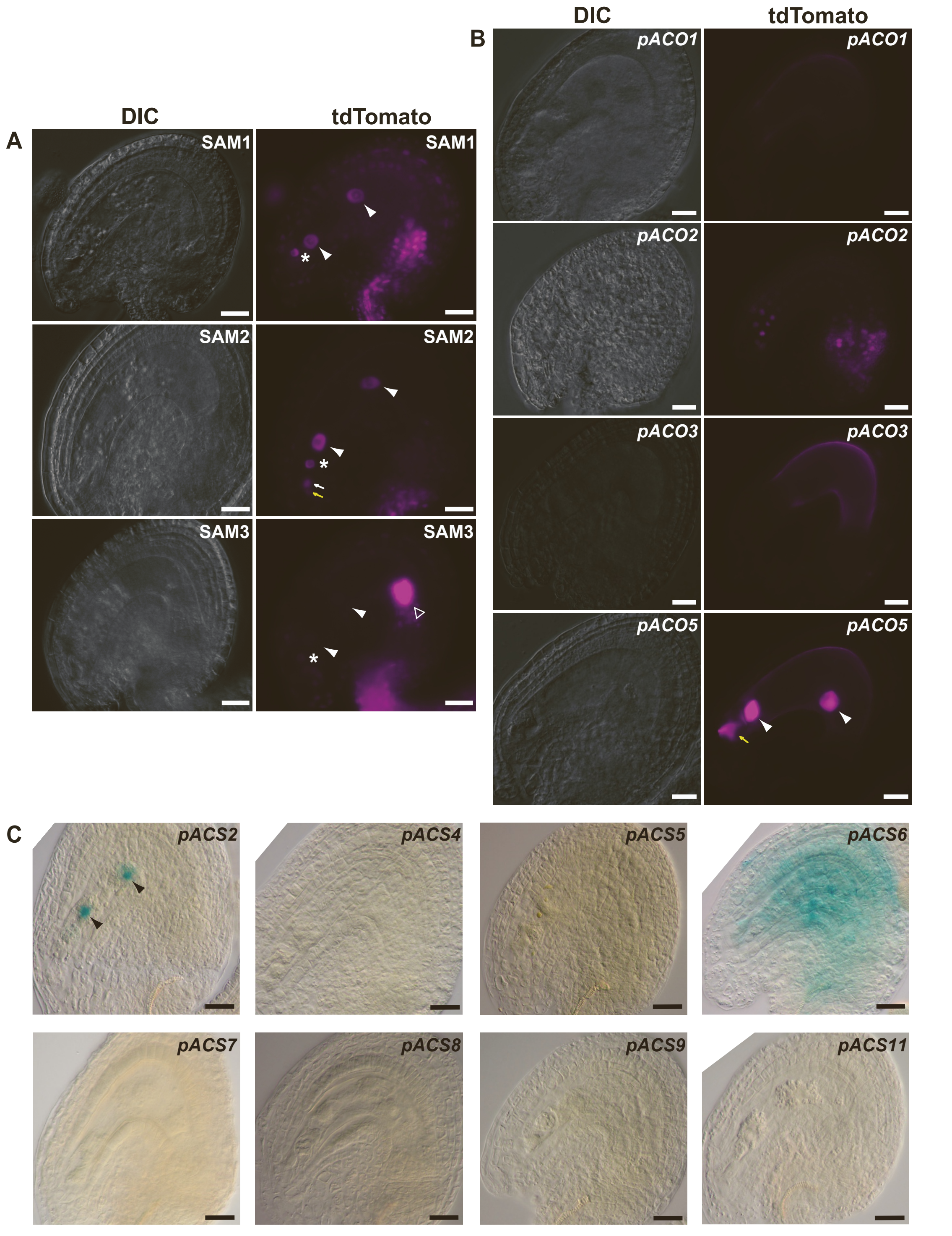

Figure S3: Expression pattern of ethylene biosynthesis genes in young seeds of wild type plants.

(A-C) Expression of *pSAMx::gSAMX_tdTomato* in young seeds of Col-0 plants (A), *pACOX::NLS_tdTomato* (B) and *pACSX::NLS_GUS* (C) in young seeds of L*er* plants two day after emasculation and one day after pollination. White arrow, persistent synergid nucleus; yellow arrow, degenerating synergid nucleus; asterisk, zygote; arrow head, endosperm nucleus; empty triangle, antipodal nuclei. Scale bar, 20µm.

A

	sterile(%)	aborted(%)	fertile(%)
WT	0,2	0,2	99,6
mat3/-	0,5	0,2	99,3
sam1sam2/-	0,5	0,0	99,5
sam1sam3/-	0,5	0,0	99,5
sam2sam3/-	0,4	0,0	99,6
acs6-1/-	0,4	0,2	99,4
acs octuple/-	49,1	14,2	36,7
aco2/-	0,8	0,4	98,9
aco2aco3/-	0,7	0,2	99,1

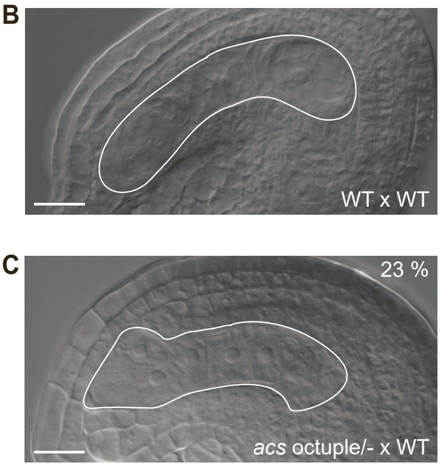

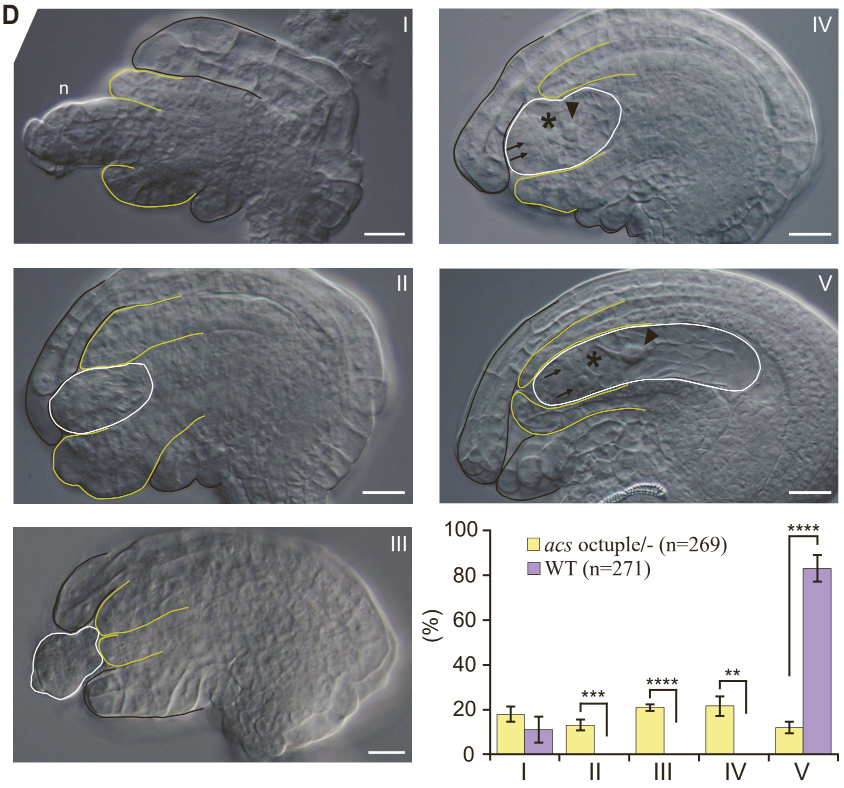

Figure S4: Female gametophyte development and integument formation are impaired in acs octuple mutants.

(A) Fertility of wild type (WT) and different ethylene synthetic mutants after selfing. (B-C) Cleared whole mounts of wild type (B) and *acs* octuple embryo sacs (C) at the four-nucleated endosperm stage. 23% of the *acs* octuple seeds at the four-nucleate endosperm stage showed abnormal embryo sac at the micropylar end (n=103). (D) Cleared whole mounts of acs octuple ovules in 2DAE carpels indicate severe developmental abnormalities. Categories are as following. (**I**): Early arrest. This category indicates the ovules in a developmental stage between around 2-IV to 3-**II** (Schneitz et al., 1995). The figure represents 2-IV staged ovule. (**II**): Ovules containing swollen integuments and abnormally developed female gametophyte (**III**): Ovules containing undeveloped integuments and expelled female gametophyte (**IV**): Ovules containing small female gametophyte. (**V**): Wild type like ovules. This category indicates fully developed ovules with mature female gametophyte. The figure represents WT ovule. Synergid nucleus, arrow; egg cell nucleus, asterisk; central cell nucleus, triangle; nucellus, n; white line, estimated female gametophyte border; black line, estimated outer integument border; yellow line, inner integument border. Scale bars, 20µm. Error bars, mean ± SEM. Statistical significance was calculated by two-tailed Student's t-test (** p < 0.01, *** p < 0.001, **** p < 0.0001).

REFERENCE

Schneitz, K., Hülskamp, M., and Pruitt, R.E. (1995). Wild-type ovule development in Arabidopsis thaliana: a light microscope study of cleared whole-mount tissue. The Plant Journal 7:731-749. https://doi.org/10.1046/-j.1365-313X.1995.07050731.x.

Supplemental Table 1: List of primers used for molecular cloning in this study

Primer name	Primer Sequence (5'-3')	Details
IE216s<br>IE216as	A<u>**GGCGCGCC**</u>TGGGCCTTATTGGCTATGTAT (**AscI**)<br>CC<u>**TTAATTAA**</u>TCTGCTACAAAGAATAGAACAAAAA (**PacI**)	Amplification of promoter *SAM1*
IE199s<br>IE218as	CC<u>**TTAATTAA**</u>ATGGAGACTTTTCTATTCACATCTGAGTC (**PacI**)<br>ATT<u>**CCTAGG**</u>AGCTTGAGGTTTGTCCCACTTGAG (**AvrII**)	Amplification of *SAM1*
IE217s<br>IE217as	A<u>**GGCGCGCC**</u>GGCACCTCCCGGTTTGTTACATT (**AscI**)<br>CC<u>**TTAATTAA**</u>TTCTTAAAGCTATAACAACAAAAAGAAAAATTGAATC (**PacI**)	Amplification of promoter *SAM2*
IE203s<br>IE219as	CC<u>**TTAATTAA**</u>ATGGAGACTTTCCTATTCACATCTGAG (**PacI**)<br>ATT<u>**CCTAGG**</u>AGCTTGAGGTTTGTCCCACTTG (**AvrII**)	Amplification of *SAM2*
IE209s<br>IE209as	A<u>**GGCGCGCC**</u>TTTTGCAGGTAATTTCTCCTTCGTTGC (**AscI**)<br>CC<u>**TTAATTAA**</u>TTTTCCGATCTGATTCACGAAAGAAACC (**PacI**)	Amplification of promoter *SAM3*
IE211s<br>IE220as	CC<u>**TTAATTAA**</u>ATGGAATCTTTTTTGTTCACATCTGAATCC (**PacI**)<br>ATT<u>**CCTAGG**</u>AGCTTGGACCTTGTTAGACTTGAG (**AvrII**)	Amplification of *SAM3*
SG12s<br>SG12as	AT<u>**GGCGCGCC**</u><u>*CTCGAG*</u>GACATGATCACTGTGAAGTCG (**AscI**-*XhoI*)<br>AT<u>**CGATCG**</u>TTGCTGTGTCAATTCTCACTTC (**PvuI**)	Amplification of promoter *ACS2*
RV283s<br>RV283as	AT<u>**GGCGCGCC**</u>AACTTAATGTTTATGTAATGATTAATATG (**AscI**)<br>AT<u>**CGATCG**</u>TTCTTTTGTTCTTGTTTTTTTTTTTTTAA (**PvuI**)	Amplification of promoter *ACS4*
RV284s<br>RV284as	AT<u>**GGCGCGCC**</u>ACCGAAATATATGGCTTCATC (**AscI**)<br>AT<u>**CGATCG**</u>TCTCTGTTTTTAAAGTCAAGAG (**PvuI**)	Amplification of promoter *ACS5*
SG37s<br>RV285as	AT<u>**GGCGCGCC**</u>AAAAATAGACCGCCTTTACAG (**AscI**)<br>AT<u>**TTAATTAA**</u>TTTTTGTTTCTTCTTTAATATAGGTTTC (**PacI**)	Amplification of promoter *ACS6*
RV274s<br>RV274as	AT<u>**GGCGCGCC**</u>ACTCACTATTAATTGCGATATGTGG (**AscI**)<br>AT<u>**CGATCG**</u>TTTTTTCTTAGAGCTTCGAACCTG (**PvuI**)	Amplification of promoter *ACS7*
SG38s<br>SG14as	AT<u>**GGCGCGCC**</u>ATATGTGTGTGTGTATTAAATTAATATGG (**AscI**)<br>AT<u>**CTCGAG**</u>TTTCTTAATTAGCTCTAGAGATAGAG (**XhoI**)	Amplification of promoter *ACS8*
SG15s<br>SG15as	AT<u>**GGCGCGCC**</u>GCAAGTTCTGTTTTCAGAAGAAG (**AscI**)<br>AT<u>**CGATCG**</u>TTTTTGATATAAAAATCAAAAAGAATGTTTGG (**PvuI**)	Amplification of promoter *ACS9*
SG17s<br>SG17as	AT<u>**GGCGCGCC**</u>GTCATTTTCACTTTTAAGATGG (**AscI**)<br>AT<u>**TTAATTAA**</u>TTTTTTTAAATGCTATAACTTGGTG (**PacI**)	Amplification of promoter *ACS11*
JH119s<br>JH119as	AT<u>**GGCGCGCC**</u>GTGTGTTAAGAACACGCGCC (**AscI**)<br>GC<u>**TTAATTAA**</u>CTCTTTTTTATTTACTTTTTCTCACACACAG (**PacI**)	Amplification of promoter *ACO1*
JH120s<br>JH120as	AT<u>**GGCGCGCC**</u>CAAACACATACAGTGCGTCGG (**AscI**)<br>GC<u>**TTAATTAA**</u>CTTTCTTTCTCTCTCTCTTCTTTGAAAG (**PacI**)	Amplification of promoter *ACO2*

JH123s<br>JH123as	AT**<u>GGCGCGCC</u>**GTGAATATATACTCTGACCCAAGGG (**AscI**)<br>GC**<u>TTAATTAA</u>**CTCTCTCTCTCTCTCTTAACTAGC (**PacI**)	Amplification of promoter *ACO3*
JH124s<br>JH124as	AT**<u>GGCGCGCC</u>**GATCATTTCCTTATGGGGTTCTG (**AscI**)<br>GC**<u>TTAATTAA</u>**TTCAGATCCGCAAAGAGAGAGAG (**PacI**)	Amplification of promoter *ACO5*
YM13-F<br>YM14-R	**<u>ATTG</u>**AATATCCCGGGGATTCATGA (**BbsI**)<br>**<u>AAAC</u>**TCATGAATCCCCGGGATATT (**BbsI**)	sgRNA of *ein3-c1*

Supplemental Table 2: List of primers used for PCR based genotyping in this study.

Primer name	Primer Sequence (5'-3')	Details
IE53s<br>RV130as	**GAACTAGT**GTTTAATGAGATGGGAATGTG (**SpeI**)<br>TTAGCAATATCAGGAAACATATGC	*ein3*[c] line confirmation (PCR product was digested by NlaIII)
IE222s	CCACTCAAGTGGGACAAACCTCAAGC	Genomic fragment of *SAM1* (IE222s+IE222as)
IE222as	CAATTTGCCAAAGATCACATTGCCCTAACTC	T-DNA verification for *sam1* (IE222as+LBa1) - (SALK-073599)
IE223s	GAATCGAATCTCTTTGGATGAGATGCGTC	Genomic fragment of *SAM2* (IE223s+IE223as)
IE223as	GGTCACCAGCTCCAATGTCTTCTGG	T-DNA verification for *sam1* (IE223as+LBa1) - (SALK-097197)
IE210s	CTGTCAAGTTGTACTCGCACGCGG	T-DNA verification for *sam3* (IE210s+LBa1) - (SALK-052289)
IE209as	CC**TTAATTAA**TTTTCCGATCTGATTCACGAAAGAAACC (**PacI**)	Genomic fragment of *SAM3* (IE210s+IE209as)
IE266s	TGAGGCCTGATGGTAAGACAC	Genomic fragment of *MAT3* (IE266s+IE266as)
IE266as	TAAAGGGACATCGACAAGTGCC	T-DNA verification for *mat3* (IE266as+LBb1.3) - (SALK-019375)
IE38s	GCAAATGAGACGATCATGTTCTG	Genomic fragment of *ACS1* (IE38s+IE38as)
IE38as	CGACGAGCCAGGAGAGAC	T-DNA verification for *acs1-1* (IE38as+NN186as)
NN186as	CCATATTGACCATCATACTCATTGC	GABI-Kat T-DNA LB primer
IE39s	GGTGGCGGACAGGTGTCGAG	T-DNA verification for *acs2-1* (IE39s+LBa1)
IE39as	CCATCCAGGTTCCGTGCAACGGAAG	Genomic fragment of *ACS2* (IE39s+IE39as)
IE41s	CGAAGAAGCCTACGAGCAAGCCAAG	T-DNA verification for *acs4-1* (IE41s+LBa1)
IE41as	CGTCTTCTTCCTCGAACCGTTTAGTC	Genomic fragment of *ACS4* (IE41s+IE41as)

RV378s	CCAGCTATGTTTCGATCTAATCGAGTCATGGTTAAC	Genomic fragment of *ACS5* (IE378s+IE378as)
RV378as	TCCATGAAACCCGGAAAACCCAGTTAGAGACTGTC	T-DNA verification for *acs5-2* (IE378as+LB1)
IE42s	GGTGGCTTTTGCAACAGAGAAGAAGCAAGATC	T-DNA verification for *acs6-1* (IE42s+LB1)
IE42as	CGATCTCCTCGATTACTTCCGCAACAC	Genomic fragment of *ACS6* (IE42s+IE42as)
IE43s	CTACAAATTGCCTTTTCTTATCGAC	FLAG T-DNA LB primer
IE44s	CGTTTGATCTTCTTGAAACTTAC	T-DNA verification for *acs7-1* (IE44s+IE43s)
IE44as	GGTATCGTACCGTCTTCTAAG	Genomic fragment of *ACS7* (IE44s+IE44as)
RV381s	GGATGGGAAGAATACGAGAAGAACCC	T-DNA verification for *acs9-1* (RV381s+LBa1)
IE45as	GGAGACTTCGCTGTTCTCGAGG	Genomic fragment of *ACS9* (RV381s+IE45as)
RV350s<br>CK138as	GACACCCCCGGCTAAGGAGACTTTCACAGGTCGTGATATG<br>GCGGATAACAATTTCACACAGGAAACAG	Verification of miRNA319a targeting ACS8 and ACS11
LB1	GCCTTTTCAGAAATGGATAAATAGCCTTGCTTCC	SAIL T-DNA LB primer
LBa1	TGGTTCACGTAGTGGGCCATCG	SALK T-DNA LB primer
LBb1.3	ATTTTGCCGATTTCGGAAC	SALK T-DNA LB primer
JH125s	CCTCAAACAGTCAAACTAAACG	T-DNA verification for *aco1* (JH125s+Lbb1.3) - (SALK-127963)
JH119as	GC**TTAATTAA**CTCTTTTTTATTTACTTTTTCTCACACACAG (**PacI**)	Genomic fragment of ACO1 (JH125s+JH119as)
AH13s	CAAAGGACCATTACAAGACATG	T-DNA verification for *aco2* (AH13s+Lbb1.3) - (SALK-027311)
AH13as	CTCTTACCAAAGTCTTTCATGG	Genomic fragment of *ACO2* (AH13s+AH13as)
AH10s	CGACGATGCTTGTCAAAACTGG	Genomic fragment of *ACO3* (AH10s+AH10as)
AH10as	CATTTGACATATCAGGGATGTCG	T-DNA verification for *aco3* (AH10as+LBa1) - (SALK-082132)

AH32s<br><br>AH32as	GATCTCGACGACGATTACAG<br><br>GAAAAATAACAGAGTCGCTTCCC	T-DNA verification for *aco4* (AH32s+LBb1.3) - (SALK-014965)<br>Genomic fragment of *ACO4* (AH32s+AH32as)
AH12s<br><br>AH14as<br><br>AH16	GTCAGAAGATGGACTAACTG<br><br>CTAGCTTCTCGCCAGAGTTC<br><br>TAACGCTGCGGACATCTACAT	Genomic fragment of *ACO5* (AH12s+AH14as)<br>T-DNA verification for *aco5-2* (AH14as+AH16) - (GK-119A07)<br>GABI-Kat T-DNA LB primer
JH235as	TCTTTGATGACCTCCTCGCC	Verification primer for C-terminally tagged tdTomato
IE214s	**A<u>GGCGCGCC</u>**GAAAACGTTTTCCTTATCTCACTTG (**AscI**)	Verification primer for *pSAM1::gSAM1_tdTomato* fragment (IE214s+JH235as)
IE207s	GATCTGCGATAGTGTGTGACTATTG	Verification primer for *pSAM2::gSAM2_tdTomato* fragment (IE207s+JH235as)
IE210s	CTGTCAAGTTGTACTCGCACGCGG	Verification primer for *pSAM3::gSAM3_tdTomato* fragment (IE210s+JH235as)
IE343s	CTTGTGATCACATGGTTGTATGG	Verification primer for *pACO1::NLS_tdTomato* fragment (IE343s+JH235as)
IE344s	CTATGACTCGCACATATTTTCTCAG	Verification primer for *pACO2::NLS_tdTomato* fragment (IE344s+JH235as)
IE345s	GCACACACACAAATACAACTTCC	Verification primer for *pACO3::NLS_tdTomato* fragment (IE345s+JH235as)
IE347s	GGTTGTACTTATGCATGCTTAGC	Verification primer for *pACO5::NLS_tdTomato* fragment (IE347s+JH235as)
IE300as	CGAACTGATCGTTAAAACTGCC	Verification primer for C-terminally tagged GUS
IE324s	GGGAGATTGATGCTAGCAAAAC	Verification primer for *pACS2::NLS_GUS* fragment (IE324s+IE300as)
IE326s	CCATGTTGGTAAATCGGATAATG	Verification primer for *pACS4::NLS_GUS* fragment (IE326s+IE300as)
IE327s	GATTTATTGCTGTATGTGTGAATGC	Verification primer for *pACS5::NLS_GUS* fragment (IE327s+IE300as)
IE328s	CAAATACTGAAATTGGACTTAATGTTATG	Verification primer for *pACS6::NLS_GUS* fragment (IE328s+IE300as)
IE329s	CGATCTCCTAAAGATGAACCAC	Verification primer for *pACS7::NLS_GUS* fragment (IE329s+IE300as)

IE330s	GCTCAATTTTGAGTGTGTTTCAG	Verification primer for *pACS8::NLS_GUS* fragment (IE330s+IE300as)
IE331s	GAGATAGGAAGAGAGATTTTCTTTAG	Verification primer for *pACS9::NLS_GUS* fragment (IE331s+IE300as)
IE333s	CACTATAGCTAATAATGTCGGGA	Verification primer for *pACS11::NLS_GUS* fragment (IE333s+IE300as)

SUPPLEMENTARY MOVIE LEGENDS

Movie S1, related to Fig. 1A: Live cell imaging of early endosperm nuclei divisions in *ein3/*+ seeds without sdn.

Pictures were taken every 10 minutes. The last frame before nuclear decondensation in the two-nucleate endosperm stage was set to 0. Frame-numbers are indicated in the video.

Movie S2, related to Fig. 1B: Live cell imaging of early endosperm nuclei divisions in *ein3/*+ seeds with sdn.

Pictures were taken every 10 minutes. The last frame before nuclear decondensation in the two-nucleate endosperm stage was set to 0. Frame-numbers are indicated in the video.
